# Salivary Gland NK Cells Are Phenotypically and Functionally Unique

**DOI:** 10.1371/journal.ppat.1001254

**Published:** 2011-01-13

**Authors:** Marlowe S. Tessmer, Emma C. Reilly, Laurent Brossay

**Affiliations:** Department of Molecular Microbiology and Immunology and Graduate Program in Pathobiology, Division of Biology and Medicine, Brown University, Providence, Rhode Island, United States of America; University of Virginia, United States of America

## Abstract

Natural killer (NK) cells and CD8^+^ T cells play vital roles in containing and eliminating systemic cytomegalovirus (CMV). However, CMV has a tropism for the salivary gland acinar epithelial cells and persists in this organ for several weeks after primary infection. Here we characterize a distinct NK cell population that resides in the salivary gland, uncommon to any described to date, expressing both mature and immature NK cell markers. Using RORγt reporter mice and nude mice, we also show that the salivary gland NK cells are not lymphoid tissue inducer NK-like cells and are not thymic derived. During the course of murine cytomegalovirus (MCMV) infection, we found that salivary gland NK cells detect the infection and acquire activation markers, but have limited capacity to produce IFN-γ and degranulate. Salivary gland NK cell effector functions are not regulated by *i*NKT or T_reg_ cells, which are mostly absent in the salivary gland. Additionally, we demonstrate that peripheral NK cells are not recruited to this organ even after the systemic infection has been controlled. Altogether, these results indicate that viral persistence and latency in the salivary glands may be due in part to the presence of unfit NK cells and the lack of recruitment of peripheral NK cells.

## Author Summary

Human cytomegalovirus (HCMV) is a herpesvirus that infects 50–95% of human populations. In immunocompetent individuals, a primary infection often goes unnoticed and when resolved by the adaptive immune response, HCMV enters into a latent phase. The natural mouse pathogen murine CMV (MCMV) is a well-characterized animal model of viral infection that results in a non-replicative, chronic infection of an immunocompetent animal. MCMV is cleared efficiently by cytotoxic lymphocytes in all organs of the infected host, except the submandibular gland (SMG) of the salivary glands where it persists for several months eventually becoming latent for the life of the host. The acute response to this virus is dependent in part on natural killer (NK) cell cytotoxicity, as animals deficient in NK cells rapidly succumb to infection. Here, we identify a distinct salivary gland resident NK cell population, which detects the infection but remains mostly hyporesponsive. Peripheral NK cells, which control infection in the spleen, are not recruited to the salivary gland. Altogether, these data imply that CMV latency in the SMG could result from inadequate NK cell responses and can potentially lead to immune intervention to reverse CMV latency.

## Introduction

Human herpesvirus 5, also known as human cytomegalovirus (HCMV), is a prototypical β-herpesvirus. HCMV infection is widespread with 50–95% of the adult population being seropositive. CMVs are opportunistic pathogens that promote their survival by exploiting a defective immune response. Primary HCMV infection is usually asymptomatic and controlled by the immune system, but is never completely cleared from the host and can cause recurrent infections especially at times of immunosuppression [1]. Consequently, CMV is a serious medical concern for organ transplant recipients, immunocompromised individuals and neonates, where CMV disease ranges from mild to fatal. In fact, CMV is one of the most serious viral complications for solid organ transplantation, is frequently associated with loss of sight in HIV patients, and is a major cause of congenital infection resulting in microcephaly, deafness, blindness and mental retardation [2].

The CMV family members have strict species specificity, precluding the use of an HCMV animal model. As a result, murine cytomegalovirus (MCMV) has been advantageous for advancing CMV immune research. Both HCMV and MCMV have 240kb double stranded DNA encoding more than 200 ORFs and show similarity in disease progression, as well as dissemination, persistence in salivary glands, and reactivation following immunosuppression [3]. Much is known about the numerous defensive strategies human and mouse CMV employ to evade immune detection [4], however, we have little understanding of how viral propensity for the epithelial cells of the salivary gland contributes to viral persistence. MCMV is cleared efficiently by cytotoxic lymphocytes in all organs of the infected host, except the submandibular gland (SMG) where it persists for several weeks to months depending on host, route of entry, and dose, eventually becoming latent for the life of the host [5], [6]. MCMV primarily replicates in the acinar glandular epithelial cells of the SMG where viral particles are first detectable around day 5 post-infection (p.i.). The SMG is considered a privileged site where dissemination of the virus to other tissues as well as transmission to naïve individuals is possible. The SMG provides a peculiar barrier where mucosal tissues are typically poised to respond in a Th2 fashion and stimulate the production of IgA, while denying access to commensal microbes and other environmental pathogens. This proclivity may contribute to the ability of CMV to take advantage of compartmental distinctions and evade the immune system in the SMG.

Natural killer (NK) cells are a widely distributed heterogenous population capable of responding to numerous pathogens and providing tumor surveillance. NK cells are thought to develop from CLPs in the bone marrow, although individual tissues contain phenotypically and functionally distinct subsets [7]. At present, it is not known whether these differences stem from developmental regulation or homing and retention signals unique to the tissue environment. However, the investigation of NK cells in mucosal tissue has gained attention recently as the frequency of NK cells compared to other lymphocytes is highest in non-lymphoid tissue. NK cell deficiency in both mice and humans results in severe viral infections. NK cells are critical to the early containment of CMV and their loss results in uncontrolled infection in both mice and humans [8], [9], [10]. Here, we investigate the phenotype of NK cells in the naïve SMG and their response to MCMV infection. The NK cell response to MCMV has been well characterized in the liver and spleen, however their contribution in the SMG has not been examined. Using the resistant C57BL/6 mouse strain, we found the resident SMG NK cell population has a unique phenotype. Importantly, during infection, the SMG NK cells acquire activation markers, yet have limited effector functions *in vivo* and *ex vivo*.

## Results

### Phenotype of resident SMG NK cells

**Figure 1:**
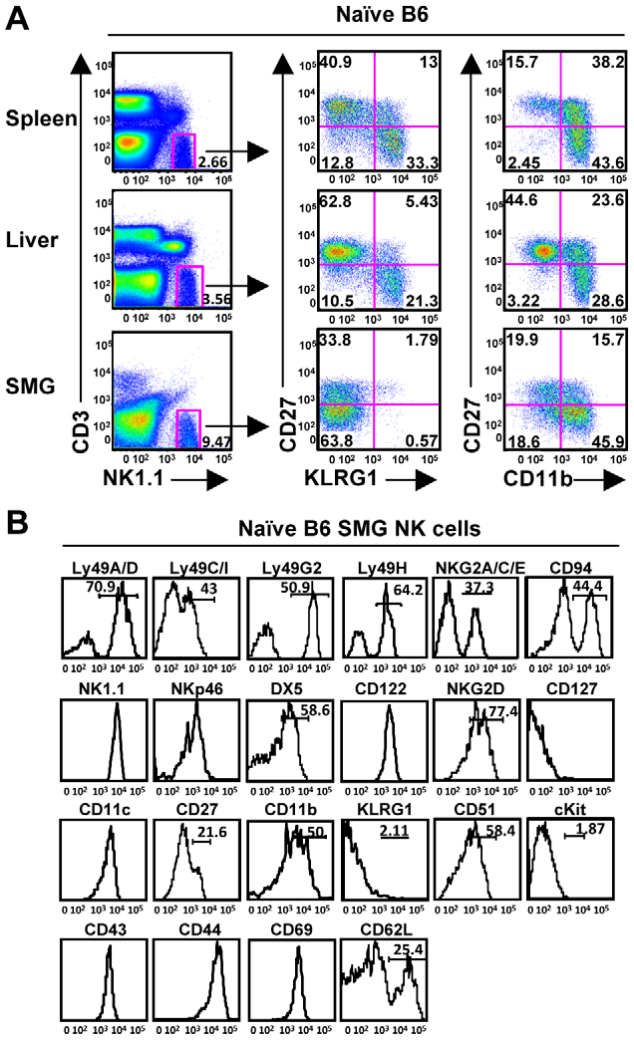
Phenotype of NK1.1^+^CD3^−^ cells in the SMG. *(A)* Naïve NK cells represent a significant population of lymphocytes within the SMG and display reduced levels of KLRG1 and CD27 compared to the spleen and liver. *(B)* Naïve NK1.1^+^CD3^−^ cells from the SMG of B6 mice were examined for other NK cell markers.

NK cells are critical for the early containment of CMV, and a population of NK cells reportedly resides in the SMG of mice and rats [11], [12]. Despite the presence of a significant NK cell population in MCMV resistant C57BL/6 mice (Figure 1A), high viral titers persist in the salivary gland for several weeks post-infection [5], [6]. We first examined the phenotype of naïve resident SMG NK cells. NK1.1^+^CD3^−^ cells compose a larger percentage of lymphocytes in the SMG vs. the liver, spleen or blood. Depending on the method of preparation, the NK cell population ranged from ∼9%–30% (Figure 1A and data not shown). These numbers are consistent with other reports of mouse and rat NK cells being more prevalent in non-lymphoid tissues, including the naïve SMG as well as during diseases like Sjogren syndrome (SS) [12], [13], [14]. Enzymatic treatment of the SMG is necessary in order to obtain adequate numbers of lymphocytes for analysis. However, we report that both Ly49H and CD27 are sensitive to both collagenase and liberase treatment (not shown). In order to account for any other changes in cell-surface markers, we compared enzymatic treatment to no treatment and found that out of all the other receptors analyzed there were no significant alterations. A modified protocol was performed which eliminated the loss of cell surface markers (see material and methods).

Expression of CD27 and CD11b subdivides mouse splenic NK cells into 4 subsets from the least mature to the most mature: CD11b^low^CD27^low^, CD11b^low^CD27^high^, CD11b^high^CD27^high^, and CD11b^high^CD27^low^ [15], [16]. KLRG1 is expressed on the most mature NK cells and is upregulated on the majority of NK cells responding to MCMV infection in the liver, spleen and blood [17], [18]. Here, we show the naïve SMG NK cells are lacking KLRG1 expression and have a significantly reduced expression of CD27, yet express mature NK cell markers CD11b, DX5, Ly49s and CD43 (Figure 1B). Furthermore, the SMG NK cells express CD51 but not CD117 (c-kit), receptors typically found on immature NK cells or NK cell precursors [18], [19]. The mean fluorescence intensity for all of these makers is provided in supplementary data (Figure S1).

The NKp46 receptor is expressed on NK cells commencing at an early stage of differentiation and has been used as a NK cell marker for mice and humans [20], [21], [22]. The SMG NK1.1^+^CD3^−^ cells express NKp46, although at slightly lower intensity than NK cells of the spleen or liver (Figure 1B and data not shown). Taken together, these data demonstrate that in naïve mice, SMG NK cells have a unique phenotype uncommon to any described to date.

### SMG NKp46^+^CD3^−^ cells are present in Rag^−/−^ mice, are not thymic derived and do not express RORγt

**Figure 2:**
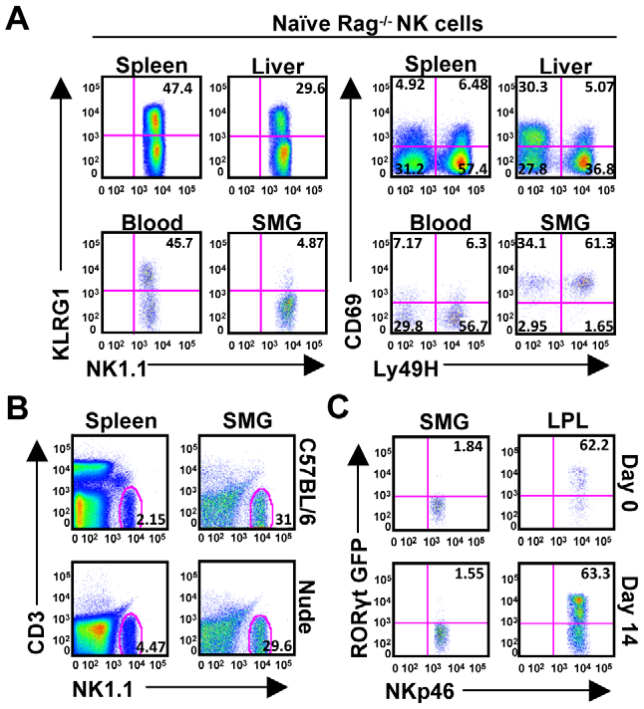
SMG NK cells are present in Rag^−/−^ mice and are not thymic or LTi derived. *(A)* Organs from naïve Rag^−/−^ mice were examined to confirm the presence of NK cells in the SMG vs. spleen, liver, and blood. NK cells from the SMG were comparably lacking cell surface KLRG1, but expressed increased levels of CD69. *(B)* Organs from naïve nude mice were examined to confirm the presence of NK cells in the SMG and spleen. *(C)* NKp46^+^CD3^−^ cells from the SMG and gut lamina propria of naïve or D14 MCMV infected RORcγt^+/GFP^ reporter mice were examined for GFP expression.

Salivary gland NK cells are highly positive for CD69, an early activation receptor. Since subsets of NKT cells [11] constitutively express CD69 and other rare T cell subsets exhibit NK cell-like features, including expression of NKp46 [23], we wanted to verify that the NK1.1^+^CD3^−^NKp46^int^ population was not a novel population of NK-like T cells. Using Rag^−/−^ mice, deficient of both T and B cells, we found an identical population of NK1.1^+^CD3^−^NKp46^int^ cells, excluding the possibility that this cell population is derived from the T cell lineage (Figure 2A). Similarly, the Rag^−/−^ NK cells are predominantly absent for the KLRG1 marker, express high levels of CD69 and consist of a distribution of Ly49H^+^ and Ly49H^−^ subsets (Figure 2A).

Despite the proximity to the thymus, the SMG NK cell phenotype is quite different from the recently reported thymic-derived NK cell subset [24]. In contrast to thymic-derived NK cells, SMG NK cells express Ly49 receptors A/D, C/I, G2 and are mostly CD11b positive (Figure 1B). Most importantly, the SMG NK cells lack CD127 (Figure 1B) and are present in nude mice (Figure 2B) ruling out a thymic contribution for SMG NK cell development.

Recently, a mucosal population of NK-like cells has been described [25], [26], [27], [28], [29]. These cells produce IL-22 and their development requires the retinoic acid receptor-related orphan receptor gamma t (RORγt) transcription factor. In order to determine whether the SMG contains these NK-like cells we used RORcγt^+/GFP^ reporter mice. Although we did find NKp46^+^/RORγt^+^ cells in the gut lamina propria as previously reported, we did not detect a significant population of these cells in the SMG (Figure 2C). Altogether, these results demonstrate that the SMG NK cells are not thymic derived and do not depend on RORγt for their development.

### SMG NK cells express activation markers during MCMV infection

**Figure 3:**
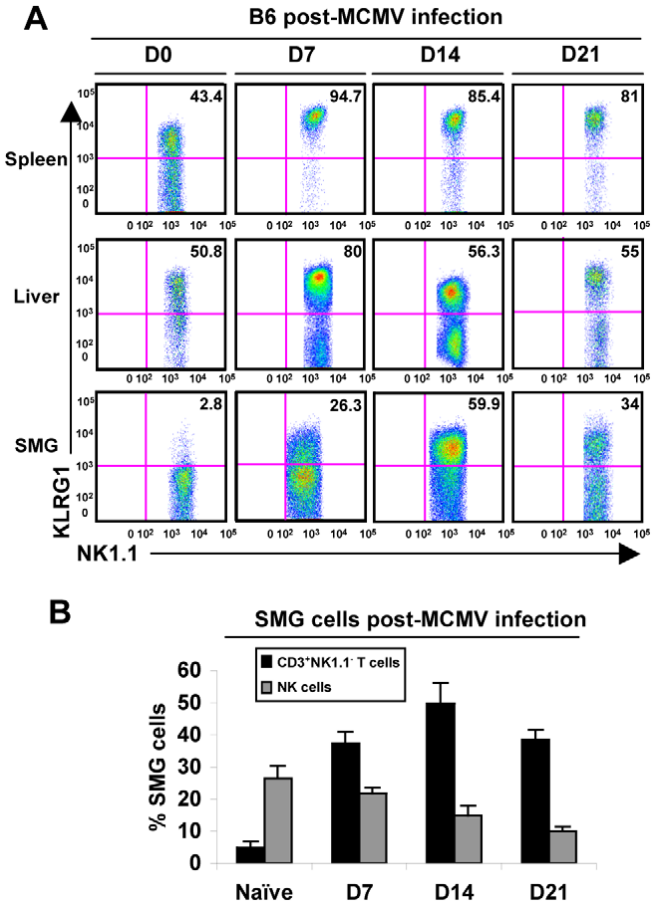
SMG NK cells become activated and induce the expression of KLRG1 during MCMV infection. *(A)* B6 mice were infected with 5×10^4^ pfu/mouse MCMV and sacrificed on D0, D7, 14 or 21 p.i. NK1.1^+^CD3^−^ cells of the SMG were compared to spleen and liver for expression of KLRG1. *(B)* The graph represents the percentage of B6 NK cells and CD8^+^ T cells in the SMG during MCMV infection at D0, 7, 14 and 21. 3–5 experiments of 2–3 mice per group is shown.

We next investigated possible changes in the SMG NK cell population during MCMV infection that could contribute to viral persistence. Mice were infected with MCMV for 7, 14 or 21 days and analyzed for expression of KLRG1 as an indicator of activation. KLRG1 expression appears on the SMG NK cells prior to D7 MCMV and by D14 is expressed at levels comparable to that of liver NK cells, a>10 fold increase (Figure 3A). Notably, Rag^−/−^ mice show a similar expression at D14 in the SMG, indicating that this phenotype occurs independently of the adaptive immune cells (Figure S2).

Additionally, CD69 expression intensifies on the NK cell population during infection (not shown), further indicating that the SMG NK cells are capable of recognizing MCMV infection. Therefore, SMG NK cells acquire activation markers during MCMV infection with a delayed kinetic compared to splenic or liver NK cells. Notably, NKp46^+^RORγt^+^ cells were not detected in the SMG during viral infection (Figure 2C).

### NK cells are not recruited to the SMG during the acute stage of MCMV

**Figure 4:**
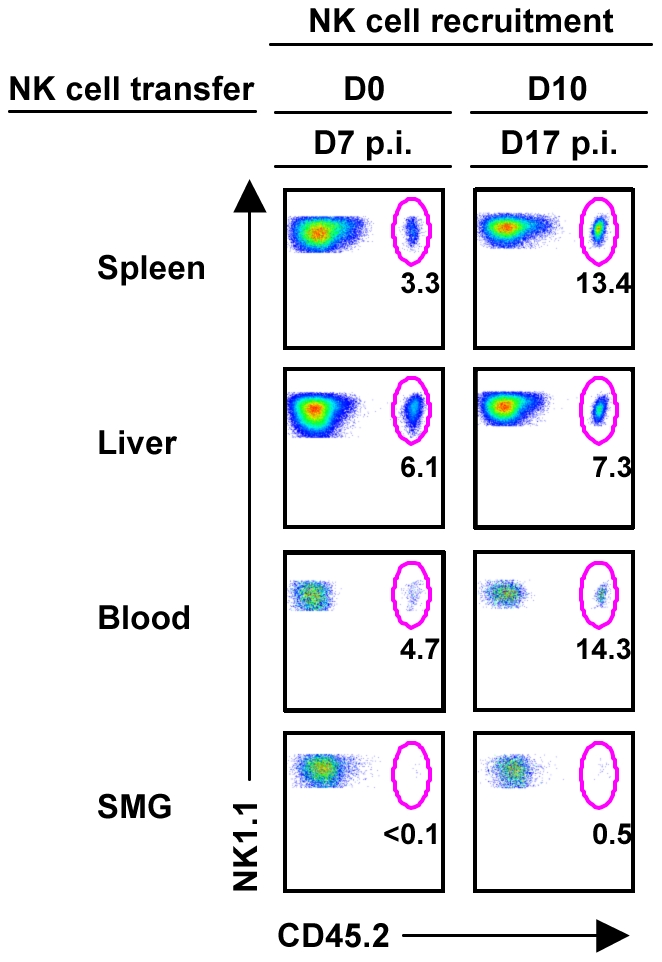
NK cells from the periphery are not recruited to the SMG during MCMV infection. B6 splenic lymphocytes were depleted of CD19^+^ and CD5^+^ cells to enrich for NK cells. Remaining cells were stained with non-reducing amounts of NK1.1 and CD3 and sorted using a FACSAria. NK1.1^+^CD3^−^ cells were of > 98% purity before i.v. injection into congenic B6 mice at 2×10^6^ cells/mouse. Recipient congenic B6 mice were either infected with 5×10^4^ pfu/mouse MCMV two hours or ten days prior to adoptive transfer. On D7 post-NK cell transfer, mice were analyzed for CD45.2^+^ NK cell trafficking. The figures represent 1 of 2 separate experiments with 2 mice per group.

During the course of infection the frequency of NK cells in the SMG decreases while the T cell population increases dramatically (Figure 3B). It is known that T cells infiltrate the salivary gland during MCMV ([11] and Figure S3A), but it has not been shown whether NK cells are actively recruited. NK cells were purified from CD45.2^+^ mice (>99%) and injected into B6 congenic recipients. To investigate whether infection alters NK cell migration, NK cells were adoptively transferred into naïve recipient mice (Figure S3B) or infected recipient mice either at the time of MCMV infection or 10 days post-infection. This time point was chosen because visceral viral replication drops below the level of detectability by D10, while MCMV replication is peaking in the SMG and typically reaches titers that are several times higher than in other organs [11]. At D7 post-MCMV infection with concurrent NK cell transfer, donor NK cells were visible in spleen, liver and blood, showing an increased percentage of infiltration in all three organs in comparison to NK cell transfer in uninfected hosts (Figure 4). At D7 post-transfer with prior MCMV infection, the NK cell percentage was further increased in spleen, liver and blood. However, under neither condition were significant numbers of transferred NK cells detectable in the SMG indicating that the resident SMG NK cells expand *in situ* but do not repopulate via a blood pathway. Therefore, it seems likely that the systemic NK cells do not receive appropriate chemotactic signals necessary to migrate to and reside in the SMG during MCMV infection, contributing to insufficient viral control in this organ.

### SMG NK cells respond weakly to MCMV *in vivo*

**Figure 5:**
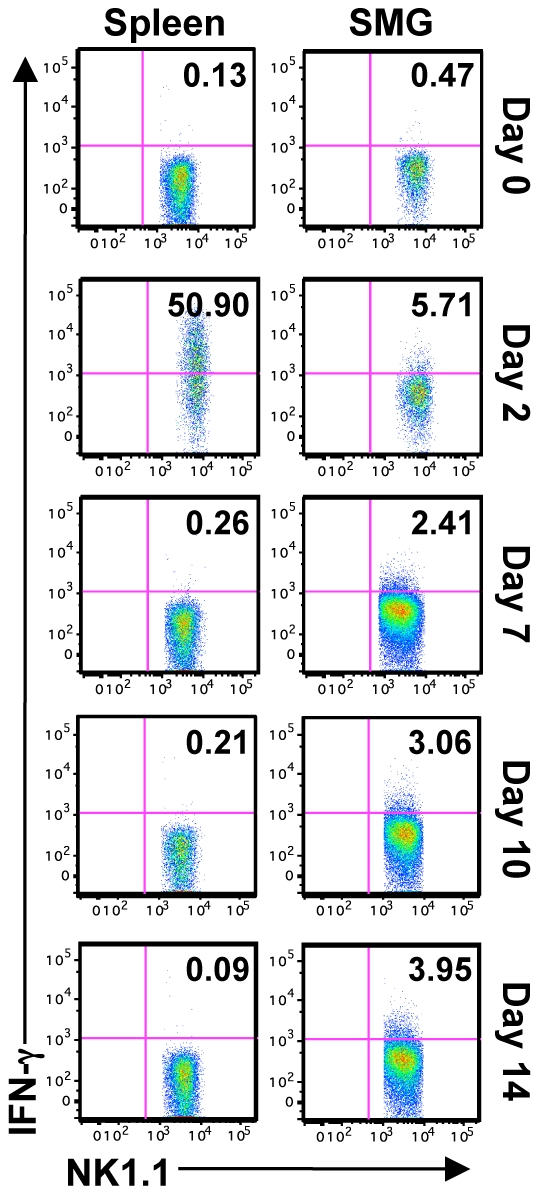
SMG NK cells are hyporesponsive *in vivo* during MCMV infection. B6 mice were infected with 5×10^4^ pfu/mouse MCMV and sacrificed on D2, 7, 10 or 14 p.i. IFN-γ production from NK1.1^+^CD3^−^ cells was determined by intracellular staining. Representative of 5 separate experiments with 2–4 mice pooled per group.

In order to study the function of SMG NK cells *in vivo*, C57BL/6 mice were infected with MCMV and NK cell IFN-γ measured at various times post-infection. The production of NK cell IFN-γ reaches its maximum in the spleen at D2 post-infection. During this time the percentage of splenic IFN-γ^+^ NK cells is around 40% ([17], [30] and Figure 5). In contrast, less than 4% of the SMG NK cells produce IFN-γ at this time point (Figure 5). At D6, D7, D9, D10, D12 and D14 post-infection, when MCMV replication in the SMG is active, no significant NK cell IFN-γ was detected in any organs (Figure 5 and Figure S4). Therefore during MCMV infection, SMG NK cells acquire activation markers such as KLRG1 with a delayed kinetic but the IFN-γ response is modest in this organ.

### SMG NK cells are hyporesponsive *ex vivo*

**Figure 6:**
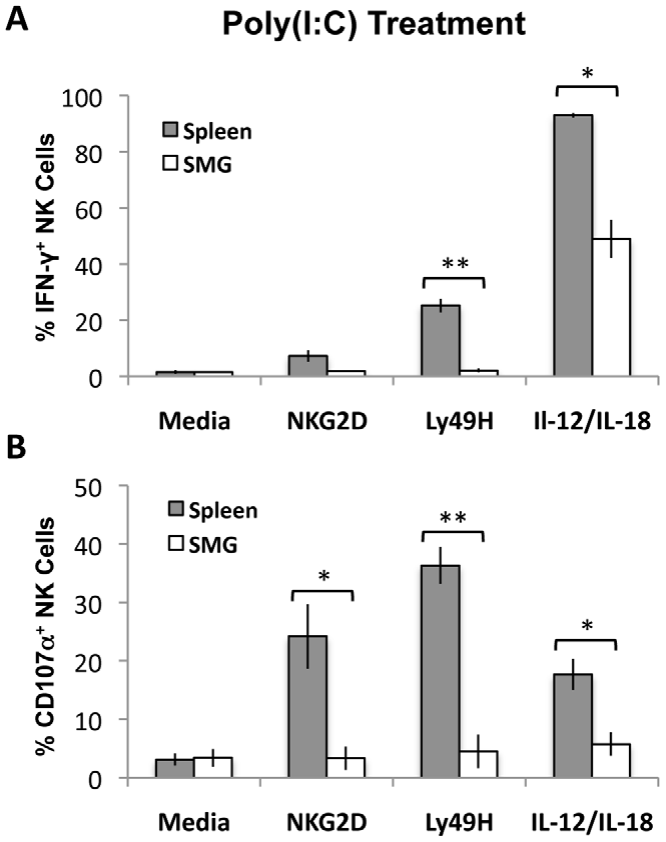
Poly(I∶C) primed SMG NK cells have impaired IFN-γ and degranulation compared with splenic NK cells. Lymphoid cells from the SMG and spleens were isolated from 24 hour Poly(I∶C) treated mice. Both organs were processed and incubated for 6 hours with no treatment, crosslinked with anti-NKG2D or anti-Ly49H, or stimulated with IL-12/IL-18. Cells were treated with Monensin and stained for IFN-γ *(A)* and CD107α *(B)* and analyzed by flow cytometry. The bar graph shows a compilation of 3 experiments with 9 mice pooled for each experiment. *p = 0.004−0.003 **p = <0.003.

Naïve NK cells do not acquire optimal effector functions unless they are primed with TLR ligands such as poly(I∶C) [31]. To measure and compare effector functions from splenic and SMG primed NK cells, B6 mice were primed *in vivo* for 24 hours with poly(I∶C). Lymphocytes from pooled salivary glands or spleens were then stimulated with anti-Ly49H, anti-NKG2D or IL-12/IL-18 for 6 hours. We found that SMG NK cells are significantly impaired in their effector functions. Poly(I∶C) primed SMG NK cells produce significantly less IFN-γ than splenic NK cells in all the conditions tested (Figure 6A and Figure S5B). In addition, their capacity to degranulate, as measured by lysosomal-associated membrane protein 1 (LAMP1), or CD107α expression, is also significantly decreased (Figure 6B and Figure S5D).

### SMG NK cell functions are impaired at the peak of MCMV replication

**Figure 7:**
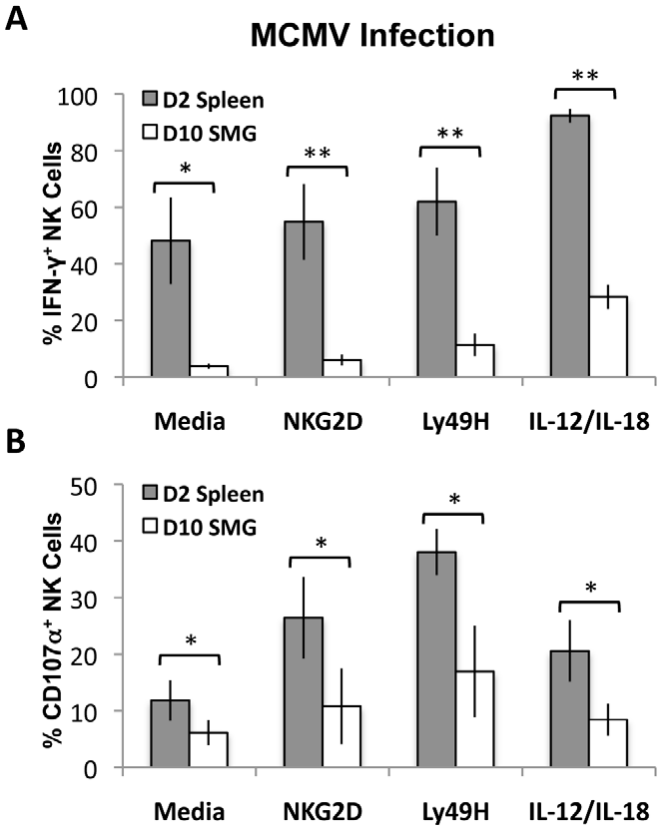
MCMV activated SMG NK cells have impaired IFN-γ and degranulation compared with splenic NK cells. Lymphoid cells from the SMG and spleens were isolated from D10 and D2 MCMV infected mice, respectively. Both organs were processed and incubated for 6 hours as described in Figure 6. Cells were treated with Monensin and stained for IFN-γ *(A)* and CD107α *(B)* and analyzed by flow cytometry. The bar graph shows a compilation of 4 experiments with 3 mice pooled for each experiment. *p = 0.03−0.003 **p = <0.003.

MCMV replication in mice is not synchronized and peaks at D2 in the spleen and D10 in the SMG. To circumvent this issue, we measured and compared NK cell effector functions in response to MCMV at the peak of replication in the respective tissues. Lymphocytes from pooled salivary glands (D10 post-infection) and spleens (D2 post-infection) were then stimulated with anti-Ly49H, anti-NKG2D or IL-12/IL-18 for 6 hours. We found that SMG NK cell production of IFN-γ is significantly decreased (Figure 7A and Figure S5A) and they have an impaired capacity to degranulate at the peak of replication (Figure 7B and Figure S5C). Altogether these data suggest that SMG NK cells are hyporesponsive upon either cytokine stimulation or activating receptor crosslinking.

### T-regs and *i*NKT cells are absent from naïve and MCMV infected SMG

**Figure 8:**
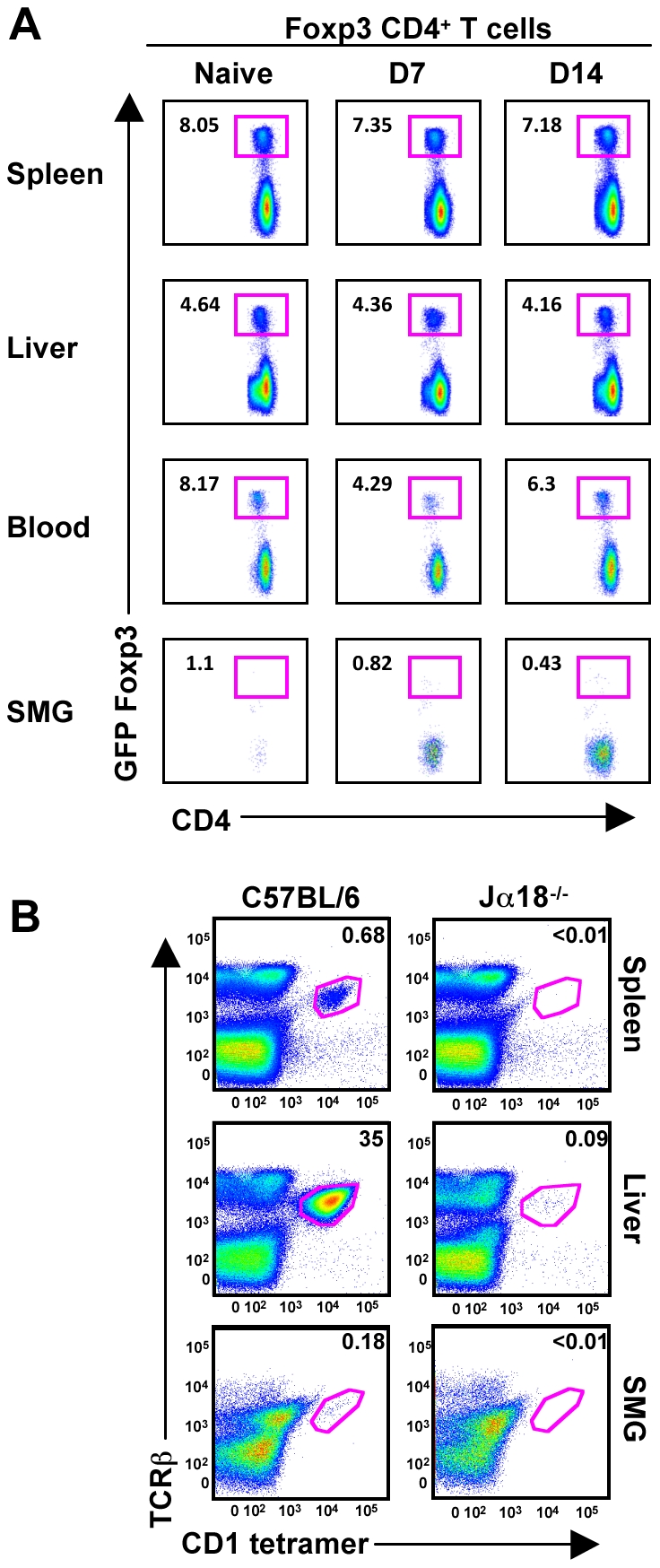
T-regs and *i*NKT cells are absent from naïve and MCMV infected SMG. *(A)* Mice expressing a GFP-Foxp3 fusion-protein reporter were infected with MCMV for the time points shown and analyzed for accumulation of T_regs_ in the spleen, liver, blood and SMG as shown by CD4^+^GFP^+^ T cells. Representative of 3 separate experiments with 3–4 mice per group. *(B)* Splenic, hepatic and SMG leukocytes were isolated from naïve B6 and Jα18 deficient mice. The *i*NKT cell compartment was analyzed by staining with TCR-β and α-GalCer-loaded CD1d tetramer. Representative of 3 separate experiments with 2–3 mice per group.

We speculated that an increase in T_regs_ might regulate the NK effector functions, influencing their unique phenotype. CD4^+^CD25^+^Foxp3^+^ T_regs_ are involved with maintaining immune homeostasis, self-tolerance and limiting tissue damage. However, using mice expressing a GFP-Foxp3 fusion-protein reporter [32], we did not detect a naïve population of resident T_regs_ nor an infiltration of GFP^+^ T cells post-MCMV infection (Figure 8A). Additionally, the naïve B6 SMG contains a population of less than 0.2% that are positive for TCRβ/CD3, NK1.1 and CD1d tetramer, indicating that *i*NKT cells are not a significant population in this organ (Figure 8B and data not shown).

## Discussion

Development of the salivary glands requires a process called branching morphogenesis to create the compact encapsulated glands with draining ducts. Branching morphogenesis is a complex developmental pathway involving cell-cell and cell-matrix interactions, cellular migration and timely proliferation as well as the appropriate response to growth factors and environmental changes. This process forms the salivary glands, lungs, kidneys and mammary glands, all places involved with potential dissemination and horizontal infection of numerous pathogens including CMV. The immune cells that organize and develop in these organs have been mostly overlooked. A need to investigate immune system recognition and responses to pathogens at barrier locations such as epithelial and mucosal tissue is becoming increasingly important with regard to understanding viral dissemination and transmission.

Significant questions involving CMV disease remain as to whether persistent viral replication in the SMG is a contributing factor to the development of latency. The existence of resident NK cells and the ability to recruit T cells to the SMG are somehow insufficient to control and eliminate virus in comparison to other organs of the host. This discrepancy implicates the SMG microenvironment and its homeostatic condition as contributing factors to viral evasion. To investigate possible reasons for viral evasion in the SMG, we examined the phenotype of the resident lymphoid cells.

We first identified a phenotypically distinct population of NK1.1^+^CD3^−^KLRG1^−^CD69^+^ cells that appears to deviate from known developmental stages. Newly described subsets of NK cells have been reported for the thymus, lymph node, lung, gut, skin, uterus, and pancreas [24], [25], [26], [27], [28], [29], [33], [34], [35]. NK cell precursors migrate to distal locations where local cytokines and receptor interactions are likely to elicit specific environmental differentiation (for review see [36], [37]). For instance, SMG expresses low amounts of MHC class I ([4]), which is required for normal NK cell maturation. Therefore, NK cell development is likely to be influenced by the unique SMG mucosal microenvironment. This is supported by a recent report from Caligiuri and colleagues who identified a novel hematopoietic precursor that predominates in the human lymph node and differentiates into CD56^bright^ NK cells [33].

We also found that SMG NK cells respond weakly to MCMV *in vivo*. Several mechanisms, not mutually exclusive, could explain this phenotype. First, low MHC class I could potentially lead to the development of hyporesponsive NK cells in the SMG, due to inefficient “licensing” or “disarming” [38], [39]. Second, inhibitory receptors could play a role in raising the threshold of activation. For instance, the KLRG1 ligands, E- and N-cadherin, are expressed in SMG [40], [41] and could potentially inhibit KLRG1^+^ NK cells during infection. Third, we noticed that SMG NK1.1^+^CD3^−^ cells express NKp46 at a lower intensity than splenic NK cells. NKp46 is a member of the Ig superfamily whose engagement initiates the activation pathway [22]. In human, a correlation between surface density of NKp46 and natural cytotoxicity has been shown [42]. In corroboration of these findings, we observed that NKp46^low^ NK cells of the SMG also have diminished cytolytic potential. Notably, SMG NK cell effector functions are also impaired after poly(I∶C) treatment suggesting their hyporesponsive status is independent of the infection. Importantly, although NK cell depletion prior to infection results in significant higher viral titer in the salivary glands [43], it has been shown that NK cell depletion at days 6 to 9 post infection has no effect on SMG MCMV titers [44] further reinforcing the findings described here.

The inability of the influxing CD8^+^ T cells and resident NK cells to provide efficient viral elimination in the SMG is puzzling. We found that T_reg_ and *i*NKT cells are unlikely to regulate NK cell functions as they are nearly absent from the SMG in both naïve and infected animals. Humphreys *et al.* found CD4^+^ T cells expressing IL-10 only localized to the SMG during MCMV infection [45]. This discovery causes speculation that the function of this population may be involved in limiting tissue injury [45]. Interestingly, although we never detected IL-10 in the serum of MCMV infected wild-type animals at any time point tested [46] we found CD3^−^NK1.1^+^GFP^+^ cells in both spleens and SMG of infected IL-10 reporter animals (Figure S6). Given the low NK cell IFN-γ production observed in SMG, it is tempting to speculate that the net outcome of the response might be in favor of the immunosuppressive function of IL-10 in this organ as suggested by others [47], [48]. IL-10^−/−^ mice show reduced serum viral titers, but greater pathology along with increased CD4^+^ T cell IFN-γ production and increased susceptibility to MCMV infection [49]. It is well known that the early inflammatory milieu, IL-12 and/or type I IFN, dictates the rate at which CD8^+^ T cells acquire memory characteristics [50], [51] and conditions NK cell proliferation and effector functions [52]. It is therefore conceivable that during MCMV infection, IL-10 not only limits Ag specific contraction resulting in increased numbers of memory CD8^+^ T cells in the SMG but also controls NK cell responses. Therefore, while MCMV utilizes an IL-10 dependent mechanism to persist in salivary gland, it could also potentially favor the development of memory CD8^+^ T cells and perhaps memory NK cells [53] preventing reactivation of the virus while limiting tissue injury. In support of this, MCMV is capable of replicating in the SMG without causing tissue damage [54], further indicating that decreased NK cell cytotoxicity could ultimately benefit the host.

## Materials and Methods

### Mice

C57BL/6, C57B6.SJL (Taconic Laboratory Animals and Services, Germantown, NY) and B6.Cg-Foxp3^tm2Tch^/J, B6.129S7-Rag1^Tm1Mom^/J, B6.129P2(Cg)-*Rorc^tm2Litt^*/J, B6.129S6-Il10^tm1Flv/J^ (Jackson Laboratory, Bar Harbor, ME) were purchased for these studies. C57BL/6NTac-Foxn1<nu>N9 (nude mice) were purchased from Taconic. B6.Ja18^−/−^ mice (kindly provided by Dr. M. Taniguchi, Riken Research Center for Allergy and Immunology, Yokohoma, Japan) were bred, crossed to B6 (>12 generations). All mice were maintained in pathogen-free breeding facilities at Brown University (Providence, RI). All mice were between 6 and 10 wks of age. Experiments were conducted in accordance with institutional guidelines for animal care.

### Infection protocols

Stocks of Smith strain MCMV salivary gland extracts or clone RVG-102 (a gift of Dr. Hamilton, Duke University) recombinant for GFP under the promoter of the immediate early gene-1 (ie-1) were prepared as previously described [17]. Infections were initiated on day 0 with 5×10^4^ plaque-forming units (PFU) of MCMV delivered i.p. For Rag^−/−^ mice 2.5×10^4^ PFU of MCMV was used.

### Adoptive transfer

Splenic lymphocytes from CD45.2^+^ B6 mice were isolated and depleted of CD5 and CD19 positive cells following AutoMACS protocol. The remaining cells were stained for CD3 and NK1.1. NK cells were sorted using a FACSAria to >98% purity. Approximately 2×10^6^ NK cells were injected i.v. into CD45.1^+^ SJL mice that had been infected with MCMV 10 days prior to transfer, 2 hours prior to transfer or left uninfected. CD8^+^ T cells were positively selected and injected i.v. into CD45.1^+^ SJL mice that were infected with MCMV 3 hours after transfer or left uninfected. Mice were sacrificed 7 days post-transfer and analyzed for NK or CD8^+^ T cell trafficking.

### NK cell effector function assay

96-well tissue culture plates were coated with anti-NKG2D or anti-Ly49H [5µg/mL]. Lymphocytes were isolated from spleen and SMG and pooled respectively. Cells were plated at 2×10^6^ cells/well and incubated with CD107α or isotype, in RPMI with Monensin (BD Biosciences) for 6 hours. As a control, cells were treated with IL-12/IL-18 [10ng/mL]. Cells were then harvested and analyzed for expression of CD107α, TCR-β or CD3, NK1.1 and intracellular IFN-γ by flow cytometry.

### Isolation of lymphocytes

To obtain splenic lymphocytes, spleens were minced, passed through nylon mesh (Tetko, Kansas City, MO), washed once in 1% PBS-serum and cell suspensions were layered on lympholyte-M (Cedarlane Laboratories Ltd., Canada). Hepatic lymphocytes were prepared by mincing and passage through a 70 mm nylon cell strainer (Falcon, Franklin Lakes, NJ). After washing 3 times in 1% PBS-serum, cell suspensions were layered on a two-step discontinuous Percoll gradient (Pharmacia Fine Chemicals, Piscataway, NJ). Salivary gland lymphocytes were prepared as described [55] with some modifications. Briefly, SMGs were removed of all lymph nodes and connective tissue, followed by mincing. Single cell dissociation was performed using one incubation with digestion medium (RPMI 1640 containing 1mg/ml of collagenase IV (Sigma), 5mM CaCl2 50µg of DNase I (Sigma) and 8% FBS) with continuous shaking at room temperature. The digestion mixture was pipetted vigorously to dissociate remaining cells. Supernatant was collected, passed through nylon mesh and the lymphocytes purified by layering on a lympholyte-M gradient. Alternatively, salivary glands were prepared, minced and incubated with liberase for 20 min. at room temperature, pipetted vigorously, washed in PBS and cell suspensions were layered on lympholyte-M. To determine possible cleavage of molecular markers by enzymatic digestion, salivary glands were also processed without digestion agents, by pressing through a 70 mm nylon cell strainer, pooled and layered on a lympholyte-M gradient. Splenocytes, hepatic lymphocytes and salivary gland lymphocytes were collected after centrifugation for 30 min at 900× g. Blood was collected by cardiac puncture and mixed with heparin sulfate. Red blood cells were lysed through incubation with NH_4_Cl for 10 min. on ice.

### Antibodies and reagents

Ly49H-APC, KLRG1-APC, CD3-PerCP-Cy7, CD3-pacific blue, CD3-APC, CD4-PE, CD8-PerCP, CD8-pacific blue, CD11b-PE, CD11b-efluor450, CD11c-APC, CD27-FITC, CD27-PE, CD43-PE, CD44-FITC, CDC45.2-FITC, CD51-PE, CD62L-APC, CD62L-PE-Cy7, CD69-PE, CD94-FITC, NKG2A/C/E-PE, CD122-PE, CD127-APC, NK1.1-APC, NK1.1-PerCP-Cy5.5, NKp46-PE, DX5-PE, CD107α-FITC, IFN-γ-PE and Ly49A/D-FITC were purchased from eBioscience (San Diego, CA). CD1d Tetramer-PE was obtained from the National Institute of Allergy and Infectious Disease MHC Tetramer Core Facility at Emory University (Atlanta, GA). NKp46-Alexa Fluor 647 was a generous gift from Dr. Vivier (Centre d'Immunologie Marseille-Luminy). Ly49H-FITC was a generous gift from Wayne Yokayama (Washington University School of Medicine, St Louis, MO) The above mentioned antibodies were used for FACS analysis in this study.

### Flow cytometric analysis

Cells were suspended in buffer comprised of PBS containing 1% FCS. Cells were first incubated with 2.4G2 mAb and stained with mAbs specific for cell surface markers for 30 min at 4°C. For intracellular staining, cells were fixed with cytofix/cytoperm and then stained for 30 min in perm/wash buffer for 30 min. Events were collected on a FACSAria, and the data was analyzed using FlowJo (Tree Star Inc.).

### Ethics statement

This study was carried out in strict accordance with the recommendations in the Guide for the Care and Use of Laboratory Animals as defined by the National Institutes of Health (PHS Assurance #A3284-01). Animal protocols were reviewed and approved by the Institutional Animal Care and Use Committee (IACUC) of Brown University. All animals were housed in a centralized and AAALAC-accredited research animal facility that is fully staffed with trained husbandry, technical, and veterinary personnel.

## Supporting Information

Figure S1 SMG NK cell marker expression. MFI of NK cell markers on SMG NK cells compared with controls.
(0.16 MB TIF)

Figure S2 SMG NK cells are present in RAG^−/−^ mice. Rag^−/−^ mice were infected with 2.5×10^4^ pfu/mouse MCMV and sacrificed on D14 p.i. SMG NK1.1^+^CD3^−^ cells were compared to spleen and liver for expression of KLRG1, CD69 and Ly49H.
(0.49 MB TIF)

Figure S3 CD8^+^ T cells infiltrate into SMG and NK cells from the periphery are not recruited to the SMG in naïve mice. *(A)* CD45.2^+^ CD8^+^ T cells were adoptively transferred into B6.SJL (CD45.1^+^) mice and evaluated for migration to different organs in naïve and D7 post-infection (gated on CD8^+^ T cells). *(B)* NK1.1^+^CD3^−^ cells prepared as described in Figure 4 were injected i.v. into congenic B6 mice at 2×10^6^ cells/mouse. On D7 post-NK cell transfer, mice were analyzed for CD45.2^+^ NK cell trafficking.
(0.34 MB TIF)

Figure S4 SMG NK cells are hyporesponsive during MCMV infection. B6 mice were infected with 5×10^4^ pfu/mouse MCMV and spleen and SMG NK1.1^+^CD3^−^ cells were assessed on D6, 9, and 12 for IFN-γ by intracellular staining. D2 spleen was used as a positive control for staining.
(0.24 MB TIF)

Figure S5 SMG NK cells are hyporesponsive after Poly(I∶C) activation and MCMV infection. Representative FACS plots of IFN-γ *(A, B)* and CD107α *(C, D)* staining of SMG and splenic NK cells after priming with MCMV *(A, C)* or Poly(I∶C) *(B, D)*.
(0.51 MB TIF)

Figure S6 SMG NK cells from IL-10 reporter mice are GFP^+^ at D10 post-infection. B6.129S6-IL10^tm1FLV^ heterozygous mice naïve, treated with 50µg/mouse CpG ODN, or infected with 5×10^4^ pfu/mouse MCMV were sacrificed at D0, D2, or D10. GFP expression on NK1.1^+^CD3^−^ cells from the spleen and SMG was determined. Representative GFP histograms of NK cells *(A)* and bar graphs including all mice evaluated *(B)* are shown.
(0.23 MB TIF)
