# Supplementary figures and images for: Salivary Gland NK Cells Are Phenotypically and Functionally Unique
Source: PLoS Pathog. 2011 Jan 13;7(1):e1001254. doi: 10.1371/journal.ppat.1001254 (PMC3020929; doi:10.1371/journal.ppat.1001254)

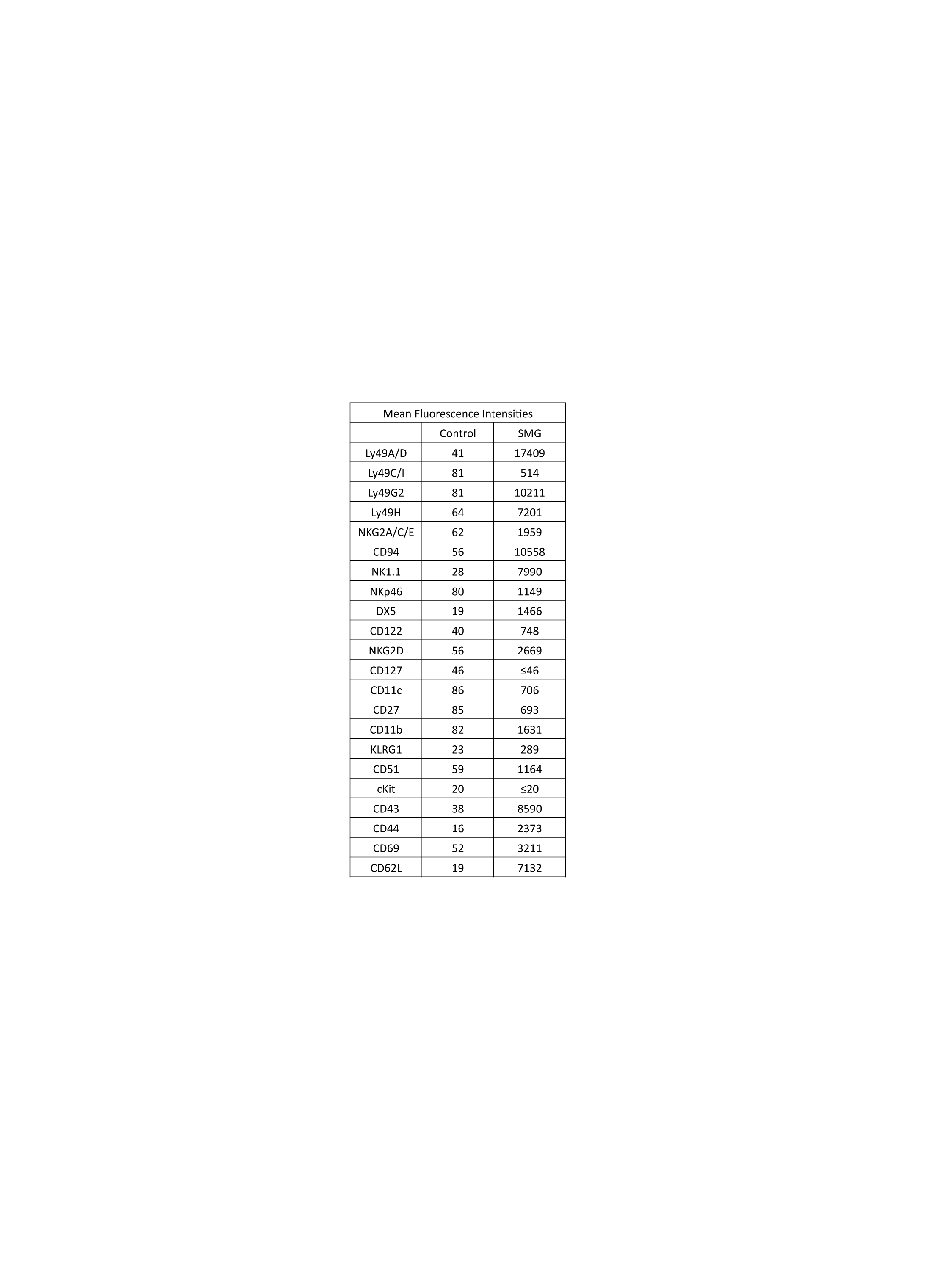

Supplement: Figure S1 — SMG NK cell marker expression. MFI of NK cell markers on SMG NK cells compared with controls. (0.16 MB TIF) [file ppat.1001254.s001.tif]

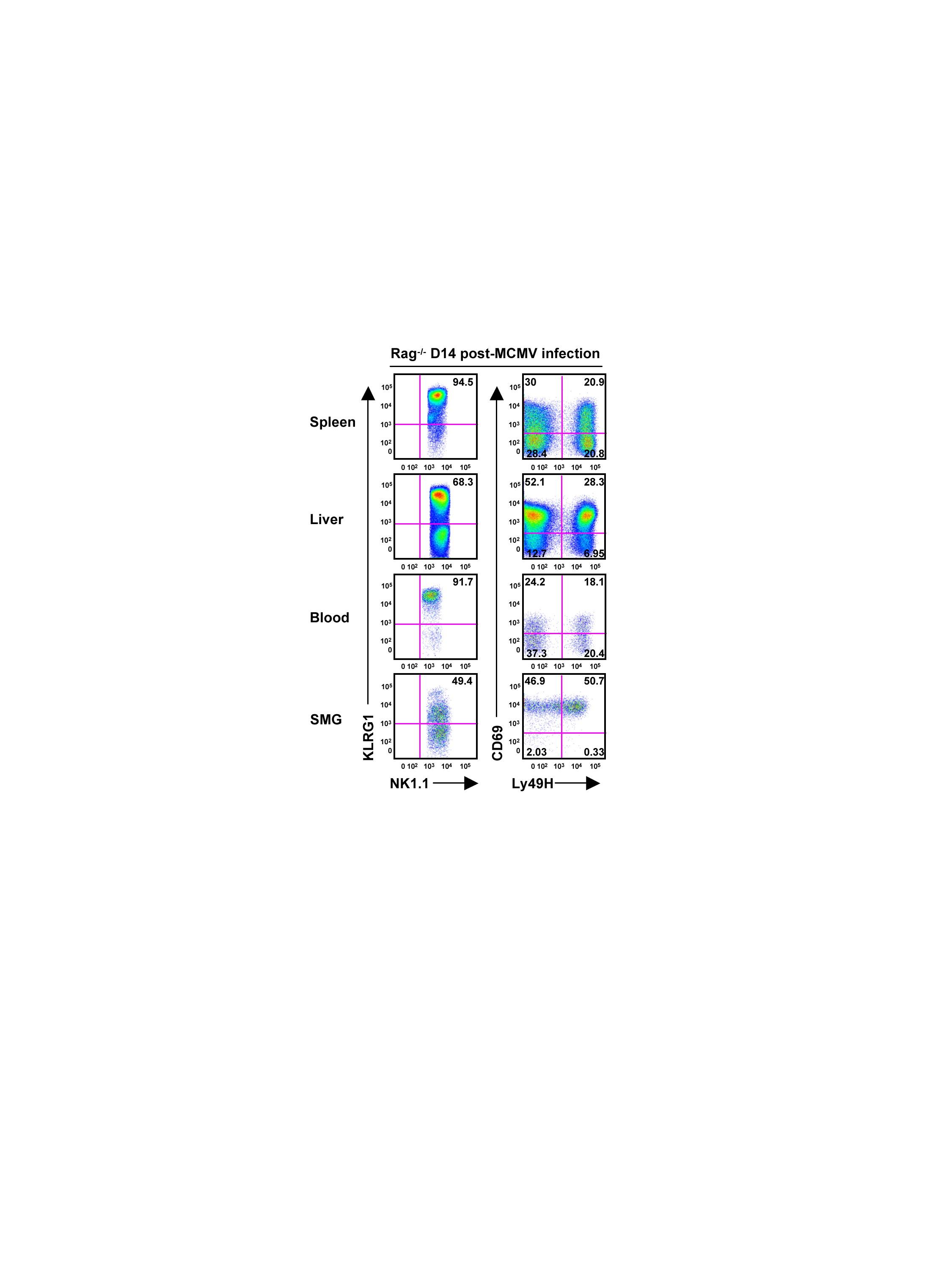

Supplement: Figure S2 — SMG NK cells are present in RAG−/− mice. Rag−/− mice were infected with 2.5×10^4^ pfu/mouse MCMV and sacrificed on D14 p.i. SMG NK1.1+CD3− cells were compared to spleen and liver for expression of KLRG1, CD69 and Ly49H. (0.49 MB TIF) [file ppat.1001254.s002.tif]

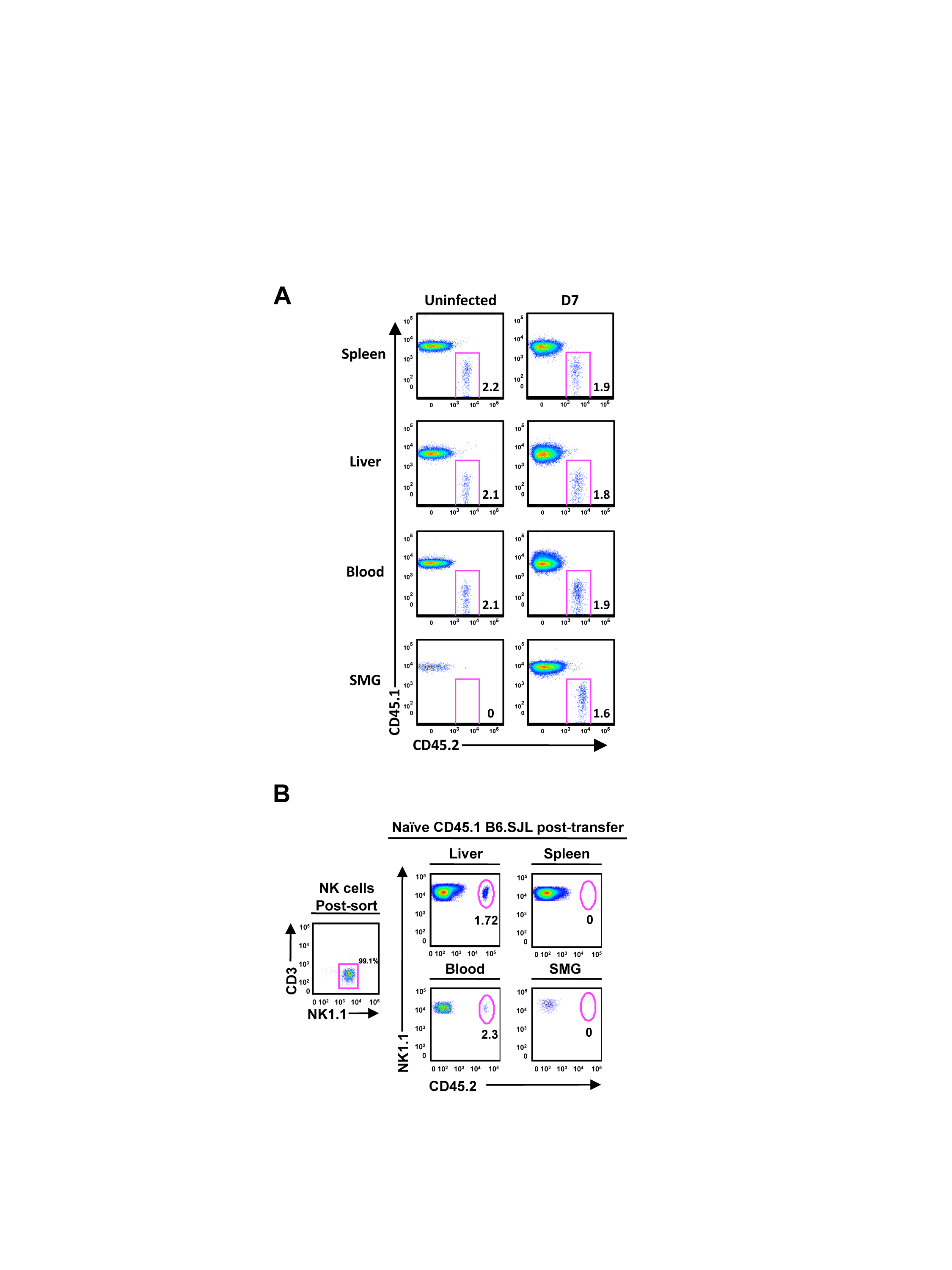

Supplement: Figure S3 — CD8+ T cells infiltrate into SMG and NK cells from the periphery are not recruited to the SMG in naïve mice. (A) CD45.2+ CD8+ T cells were adoptively transferred into B6.SJL (CD45.1+) mice and evaluated for migration to different organs in naïve and D7 post-infection (gated on CD8+ T cells). (B) NK1.1+CD3− cells prepared as described in Figure 4 were injected i.v. into congenic B6 mice at 2×10^6^ cells/mouse. On D7 post-NK cell transfer, mice were analyzed for CD45.2+ NK cell trafficking. (0.34 MB TIF) [file ppat.1001254.s003.tif]

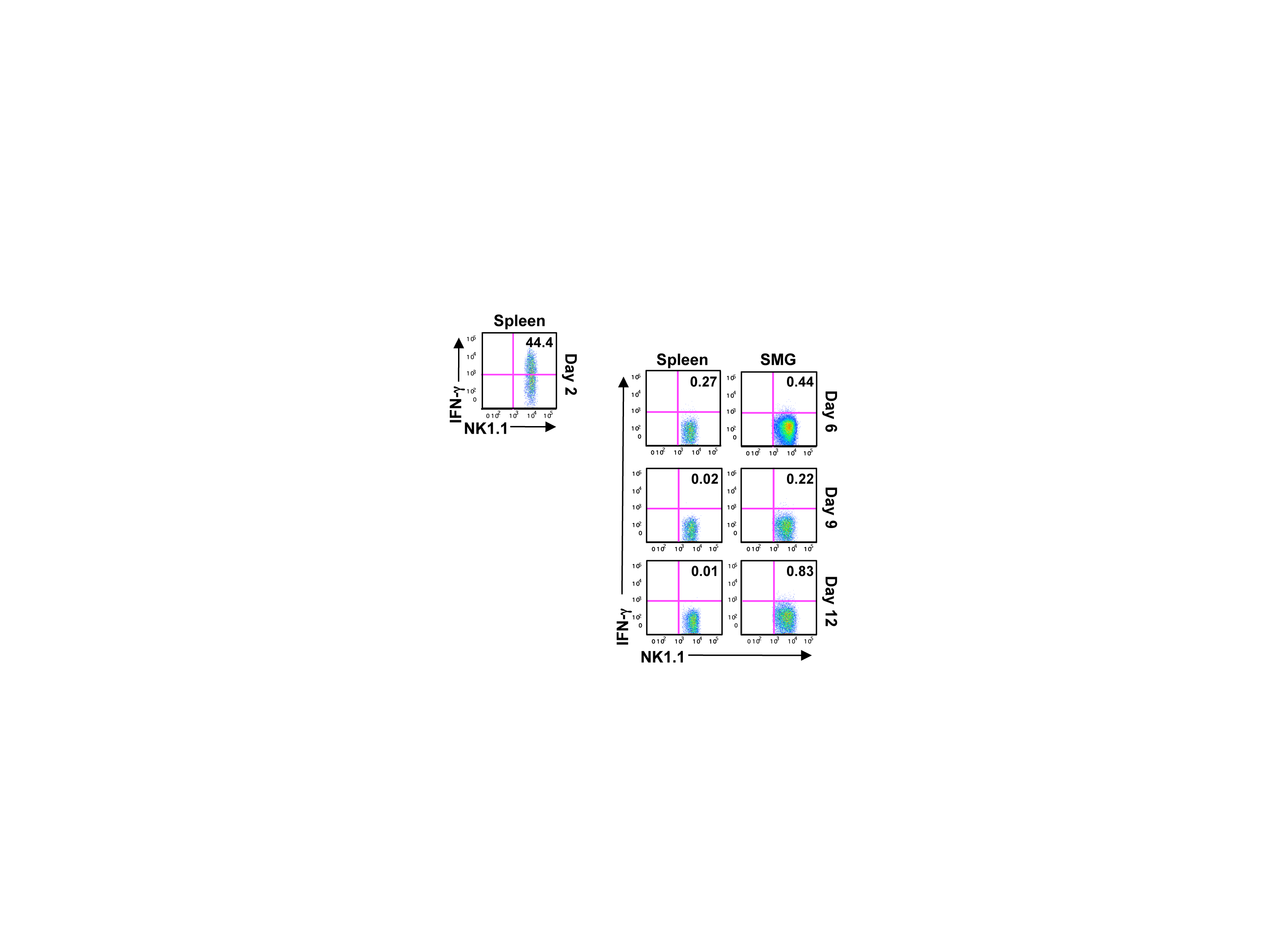

Supplement: Figure S4 — SMG NK cells are hyporesponsive during MCMV infection. B6 mice were infected with 5×10^4^ pfu/mouse MCMV and spleen and SMG NK1.1+CD3− cells were assessed on D6, 9, and 12 for IFN-γ by intracellular staining. D2 spleen was used as a positive control for staining. (0.24 MB TIF) [file ppat.1001254.s004.tif]

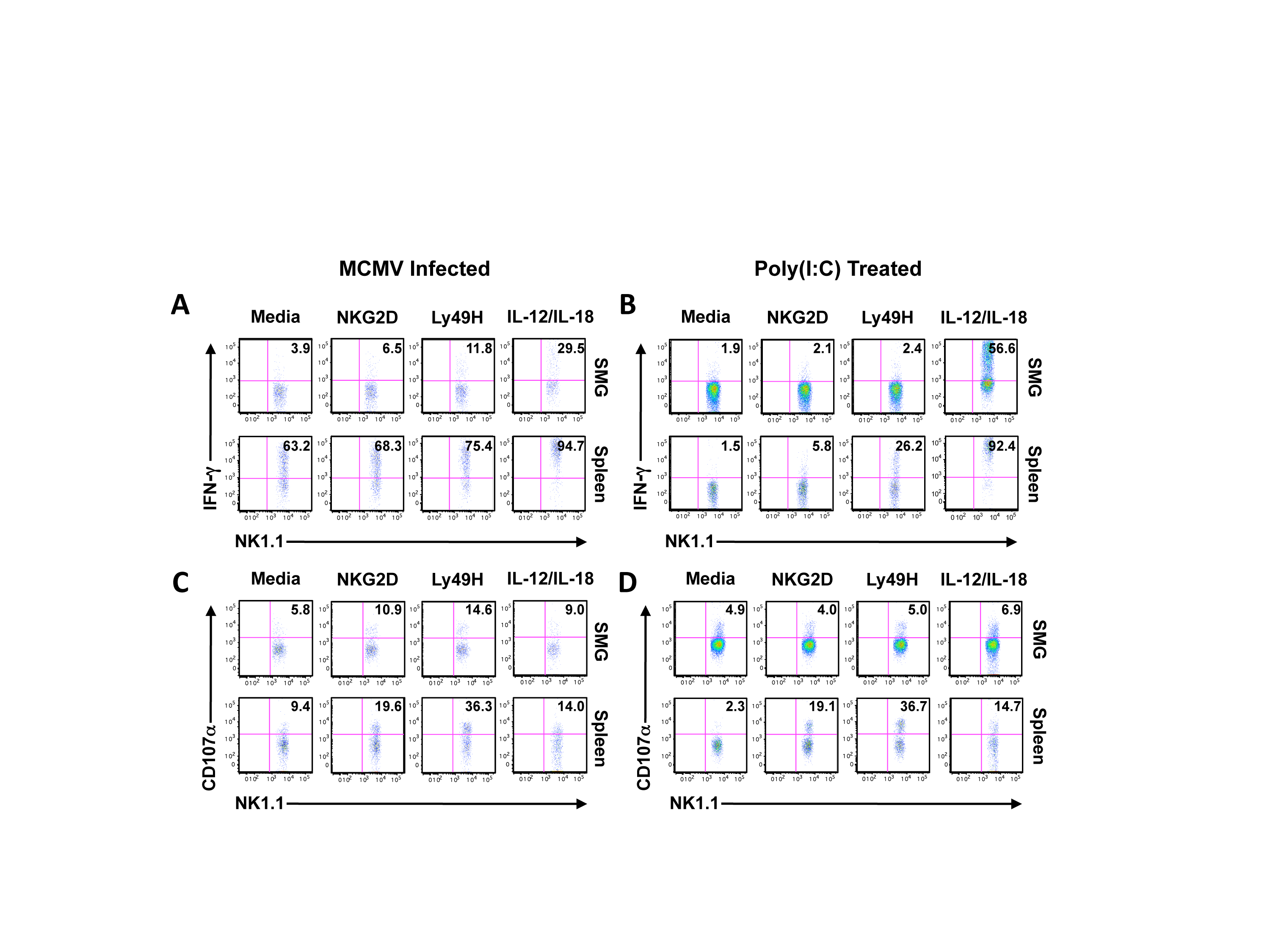

Supplement: Figure S5 — SMG NK cells are hyporesponsive after Poly(I∶C) activation and MCMV infection. Representative FACS plots of IFN-γ (A, B) and CD107α (C, D) staining of SMG and splenic NK cells after priming with MCMV (A, C) or Poly(I∶C) (B, D). (0.51 MB TIF) [file ppat.1001254.s005.tif]

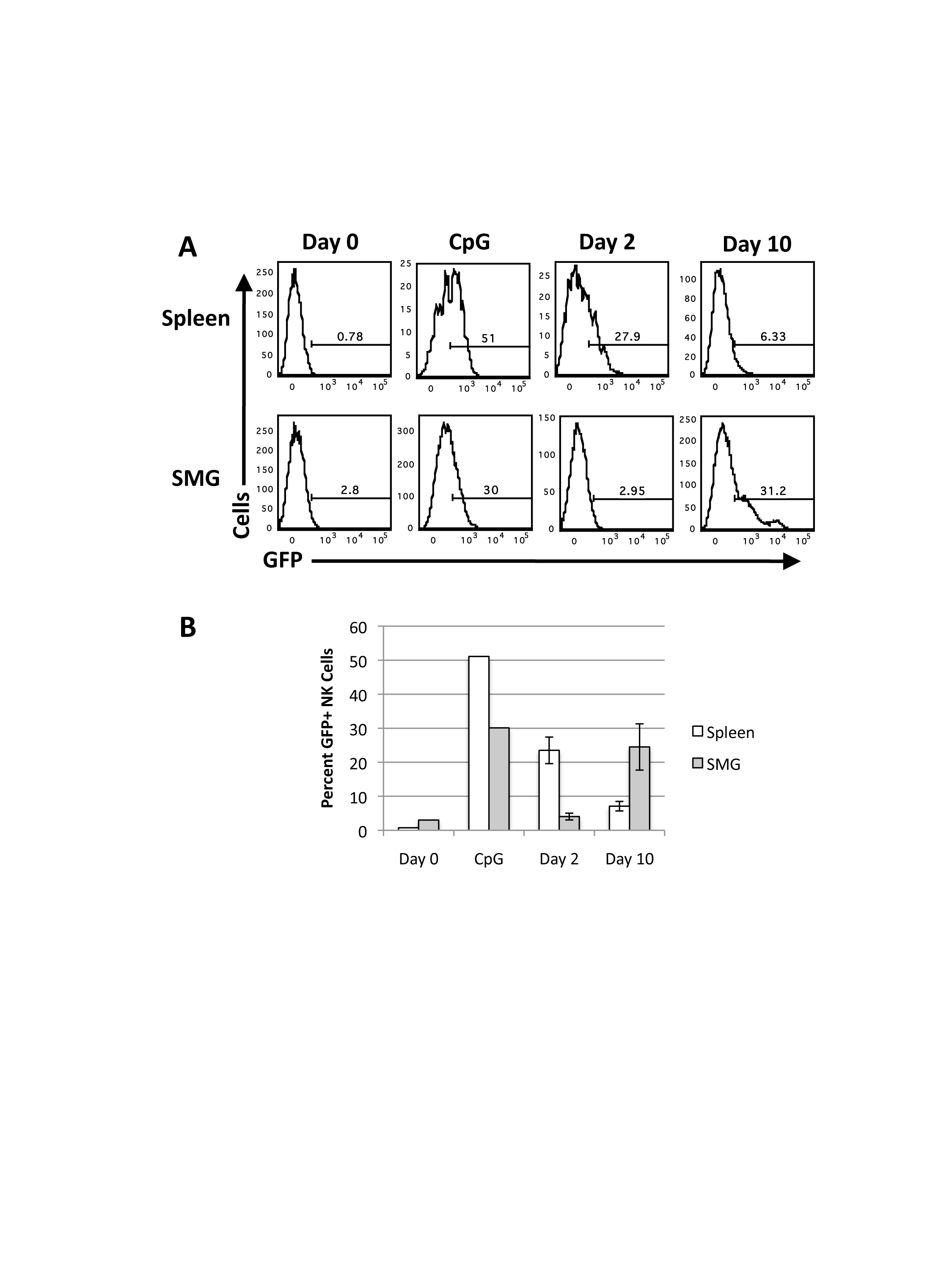

Supplement: Figure S6 — SMG NK cells from IL-10 reporter mice are GFP+ at D10 post-infection. B6.129S6-IL10tm1FLV heterozygous mice naïve, treated with 50µg/mouse CpG ODN, or infected with 5×10^4^ pfu/mouse MCMV were sacrificed at D0, D2, or D10. GFP expression on NK1.1+CD3− cells from the spleen and SMG was determined. Representative GFP histograms of NK cells (A) and bar graphs including all mice evaluated (B) are shown. (0.23 MB TIF) [file ppat.1001254.s006.tif]
